# Pathological conversion of regulatory T cells is associated with loss of allotolerance

**DOI:** 10.1038/s41598-018-25384-x

**Published:** 2018-05-04

**Authors:** Jing Hua, Takenori Inomata, Yihe Chen, William Foulsham, William Stevenson, Tina Shiang, Jeffrey A. Bluestone, Reza Dana

**Affiliations:** 1 Schepens Eye Research Institute, Massachusetts Eye and Ear Infirmary, Department of Ophthalmology, Harvard Medical School, Boston, MA USA; 2 Diabetes Center, University of California San Francisco, San Francisco, CA USA

## Abstract

CD4^+^CD25^+^Foxp3^+^ Regulatory T cells (Tregs) play a critical role in immune tolerance. The plasticity and functional adaptability of Tregs in an inflammatory microenvironment has been demonstrated in autoimmunity. Here, using a double transgenic mouse model that permits Foxp3 lineage tracing, we investigated the phenotypic plasticity of Foxp3^+^ Tregs in a well-characterized murine model of corneal transplantation. In order to subvert the normal immune privilege of the cornea and foster an inflammatory milieu, host mice were exposed to desiccating stress prior to transplantation. Treg frequencies and function were decreased following desiccating stress, and this corresponded to decreased graft survival. A fraction of Tregs converted to IL-17^+^ or IFNγ^+^ ‘exFoxp3’ T cells that were phenotypically indistinguishable from effector Th17 or Th1 cells, respectively. We investigated how Foxp3 expression is modulated in different Treg subsets, demonstrating that neuropilin-1^−^ peripherally-derived Tregs are particularly susceptible to conversion to IL-17^+^/IFNγ^+^ exFoxp3 cells in response to cues from their microenvironment. Finally, we show that IL-6 and IL-23 are implicated in the conversion of Tregs to exFoxp3 cells. This report demonstrates that the pathological conversion of Tregs contributes to the loss of corneal immune privilege.

## Introduction

CD4^+^CD25^+^Foxp3^+^ regulatory T cells (Tregs) play a critical role in mediating immunological self-tolerance. Thymus-derived tTregs, and their peripherally induced counterparts pTregs, provide essential suppressive mechanisms against deleterious immune-mediated inflammation. These specialized T cell subsets are clonal groupings within a much larger, heterogeneous population of CD4^+^ T cells – including Th1, Th2 and Th17 cells. Previously believed to perform discrete and permanent functions defined by specific cytokine profiles, there is mounting evidence that these CD4^+^ T cell subsets exhibit marked phenotypic plasticity, repolarizing towards mixed or alternative fates according to cues from their microenvironments^1,2^.

Treg plasticity has been widely observed in autoimmune conditions, such as type 1 diabetes, juvenile arthritis and multiple sclerosis^1^. Decreased stability of Foxp3 expression and increased proportions of Th1-like IFNγ^+^ Tregs have been found in these conditions^3–5^. The reprogramming of Tregs towards a memory T cell phenotype has been shown to promote inflammation in autoimmune disease^6^. However, there is limited knowledge about Treg plasticity in the context of transplantation.

Tregs play an essential role in the induction and maintenance of tolerance to alloantigens^7,8^. Tregs promote graft acceptance by suppressing host immunity against transplants. Levels of Foxp3 expression have been shown to be vitally important for the suppressor function of Tregs^9,10^. In a murine model of corneal transplantation, Foxp3 expression by Tregs has been demonstrated to be more reflective of allograft outcome than Treg absolute frequencies^11^. Still, we do not know whether the inflammatory milieu following tissue grafting provokes reprogramming of Tregs; and, if so, whether loss of Foxp3 expression can cause allorejection.

In this study, using a double transgenic mouse model that permits Foxp3 lineage tracing, we investigated the phenotypic plasticity of Foxp3^+^ Tregs in a well-characterized murine model of corneal transplantation^12–14^. We demonstrate that inflammation in the ocular tissue leads to the loss of Foxp3 in Tregs, and their conversion to ‘exFoxp3 cells’, which express the pro-inflammatory cytokines IL-17 and/or IFN-γ. Through adoptive transfer of Treg subsets, we show that neuropilin-1^−^ pTregs are highly susceptible to environmental cues that promote their conversion to exFoxp3 cells. These results provide evidence that pathological conversion of Tregs contributes to the loss of corneal immune privilege and allograft rejection.

## Results

### Dry eye disease in corneal transplant recipients limits allograft survival, and decreases the frequencies and function of Tregs

**Figure 1:**
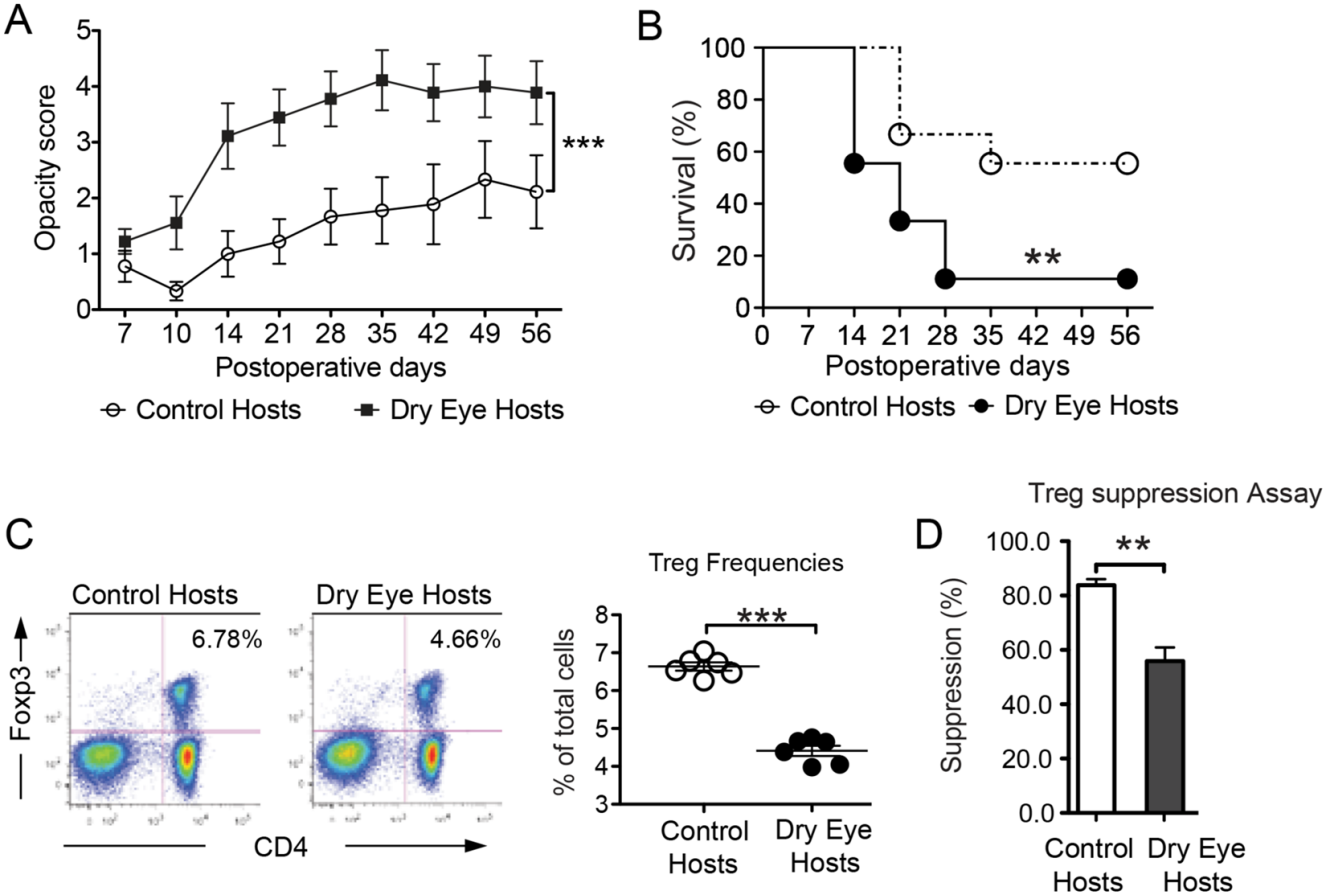
Dry eye disease reduces graft survival and Treg function after allograft survival. Healthy corneal grafts were transplanted on normal control hosts or dry eye (DED) hosts. (**A**) Corneal graft opacity (***p < 0.001) and (**B**) graft survival were assessed for 8 weeks (n = 10/group, **p < 0.01). Draining lymph nodes were harvested 2 weeks post-transplantation. Single cell suspensions were analyzed by flow cytometry. (**C**) Frequencies of CD4^+^Foxp3^+^ Tregs from control vs. DED hosts was determined using flow cytometry (n = 5 and ***p < 0.001) (**D**) Treg suppression assay (n = 6) was performed using CD4^+^CD25^−^ responders from rejected control hosts, and CD4^+^CD25^+^Tregs from control and dry eye hosts (**p < 0.01). Data is presented as mean ± SEM (error bar).

In corneal transplantation, completely mismatched allografts have a long-term survival rate of 50% without any local or systemic treatment. However, local inflammation at the transplant site (of which dry eye disease is the most common cause^15^) often jeopardizes graft survival^16^. We first performed allogeneic corneal transplantation in healthy or DED recipients. Our results demonstrated that induction of DED in graft recipients before performing corneal transplantation resulted in significantly higher graft opacity scores compared to healthy hosts (Fig. 1A). Furthermore, graft survival decreased from 60% in healthy recipients to 10% in dry eye hosts at week 8 after transplantation (Fig. 1B). Next, we analyzed the frequencies of CD4^+^CD25^+^ Tregs and the capacity of Tregs to suppress CD4^+^ CD25^−^ T cell proliferation in dry eye and healthy graft recipients, and found that both frequencies (Fig. 1C) and suppressive function (Fig. 1D) of Tregs were significantly suppressed in hosts with dry eye compared to healthy controls.

### Dry eye disease in corneal transplant recipients increases frequencies of exFoxp3 cells

It has been shown that inflammation affects Foxp3 expression in Tregs, and therefore conveys their lineage and functional instability^6^. To investigate Treg plasticity in corneal transplantation, we employed transgenic reporter mice that carry a Foxp3 lineage reporter gene construct^6,17^, in which we could trace Foxp3 expression and its loss in Tregs. In these mice, Tregs that express or used to express Foxp3 are RFP^+^. Tregs that currently express Foxp3 are GFP^+^, but loss of Foxp3 expression results in these cells becoming GFP^−^. Thus, these ‘exFoxp3 cells’ can easily be distinguished from functional Tregs (Tregs = GFP^+^RFP^+^, exFoxp3 cells = GFP^−^RFP^+^).

**Figure 2:**
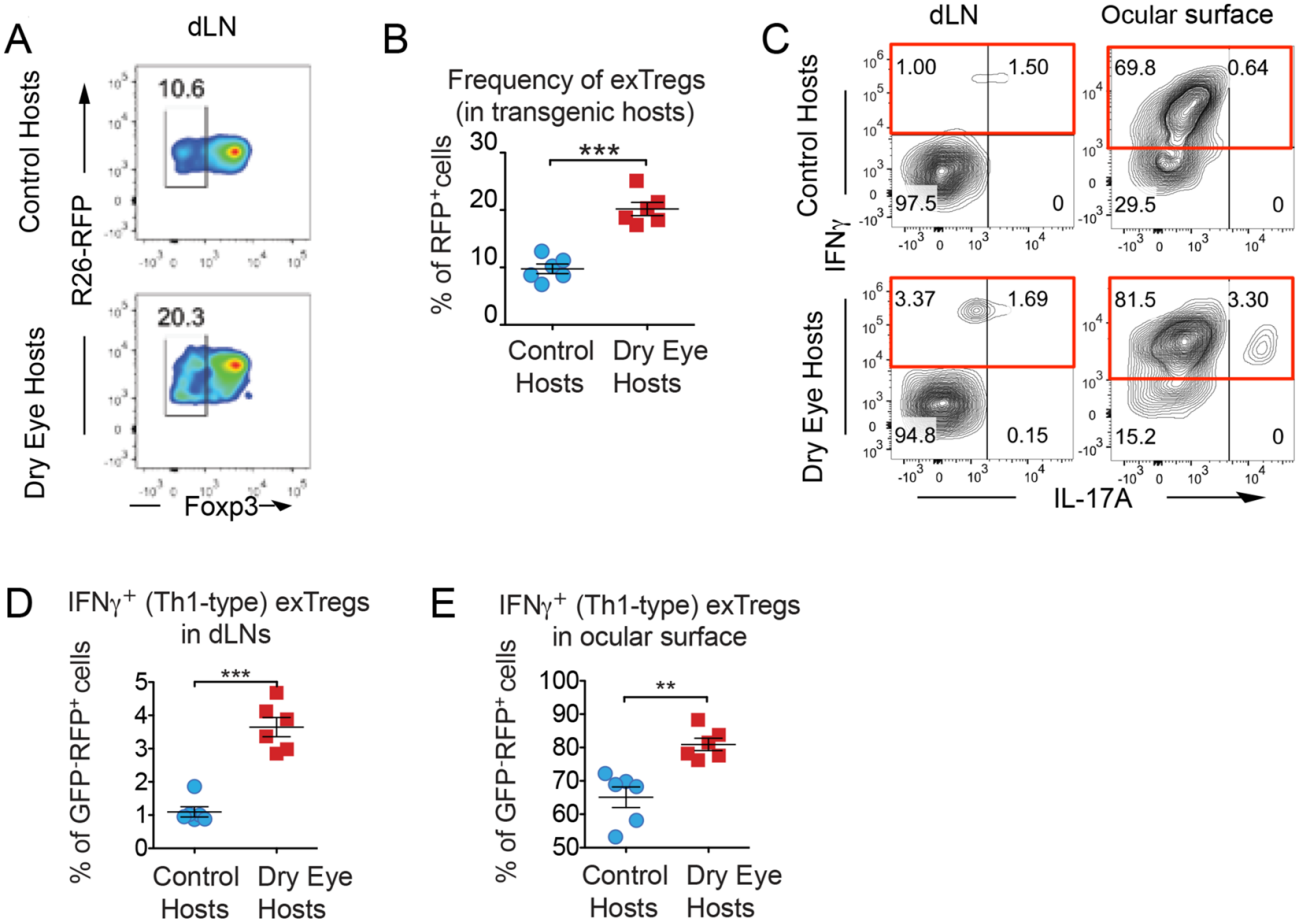
Dry eye disease promotes loss of Foxp3 expression by Tregs following corneal transplantation. Foxp3-GFP-CreXR26-RFP transgenic mice were used to trace Foxp3 expression after transplantation. Healthy corneal grafts were transplanted onto transgenic naïve control hosts or dry eye (DED) hosts. Draining lymph nodes were harvested 2 weeks post-transplantation. Single cell suspensions were analyzed by flow cytometry. (**A**) Representative flow cytometry plots and (**B**) statistical analysis of flow cytometry data showing frequencies of exFoxp3 cells in Foxp3-GFPXR26-RFP control and DED hosts (n = 6/group, ***p < 0.001). (**C**) Representative flow cytometry plots and (**D, E**) statistical analyses of flow cytometry data showing frequencies of exFoxp3 cells that express IFNγ (***p < 0.001) and IL-17 (**p < 0.01) in Foxp3-GFPXR26-RFP control and DED hosts (n = 6/group). Data is presented as mean ± SEM (error bar).

We performed corneal transplantation in healthy and DED hosts and analyzed their frequencies of GFP^−^RFP^+^ exFoxp3 cells in draining lymph nodes (dLN) at 2 weeks following transplantation. Our previous work has indicated that this is the optimum time point to evaluate the alloimmune response prior to the onset of higher rejection frequencies, which occur at week 3^11,13,14,16,18^. Importantly, although we did not observe clinically visible rejection at week 2, we found significantly higher exFoxp3 frequencies in dLN of dry eye hosts compared to healthy recipients (Fig. 2A and B). Our analysis of the inflammatory cytokine expression by these exFoxp3 cells revealed increased frequencies of IFN-γ^+^ exFoxp3 cells in both dLN and ocular surface in DED hosts relative to control recipients (Fig. 2C,D and E). IFN-γ-producing CD4^+^ T cells (Th1 cells) are the principal effector cells in corneal allograft rejection^19^; thus, the observed higher frequencies of IFN-γ^+^ exFoxp3 cells in DED hosts may contribute to higher graft rejection rates in these recipients.

### Adoptively transferred pTregs are susceptible to conversion to exFoxp3 cells in DED hosts and fail to improve graft survival

**Figure 3:**
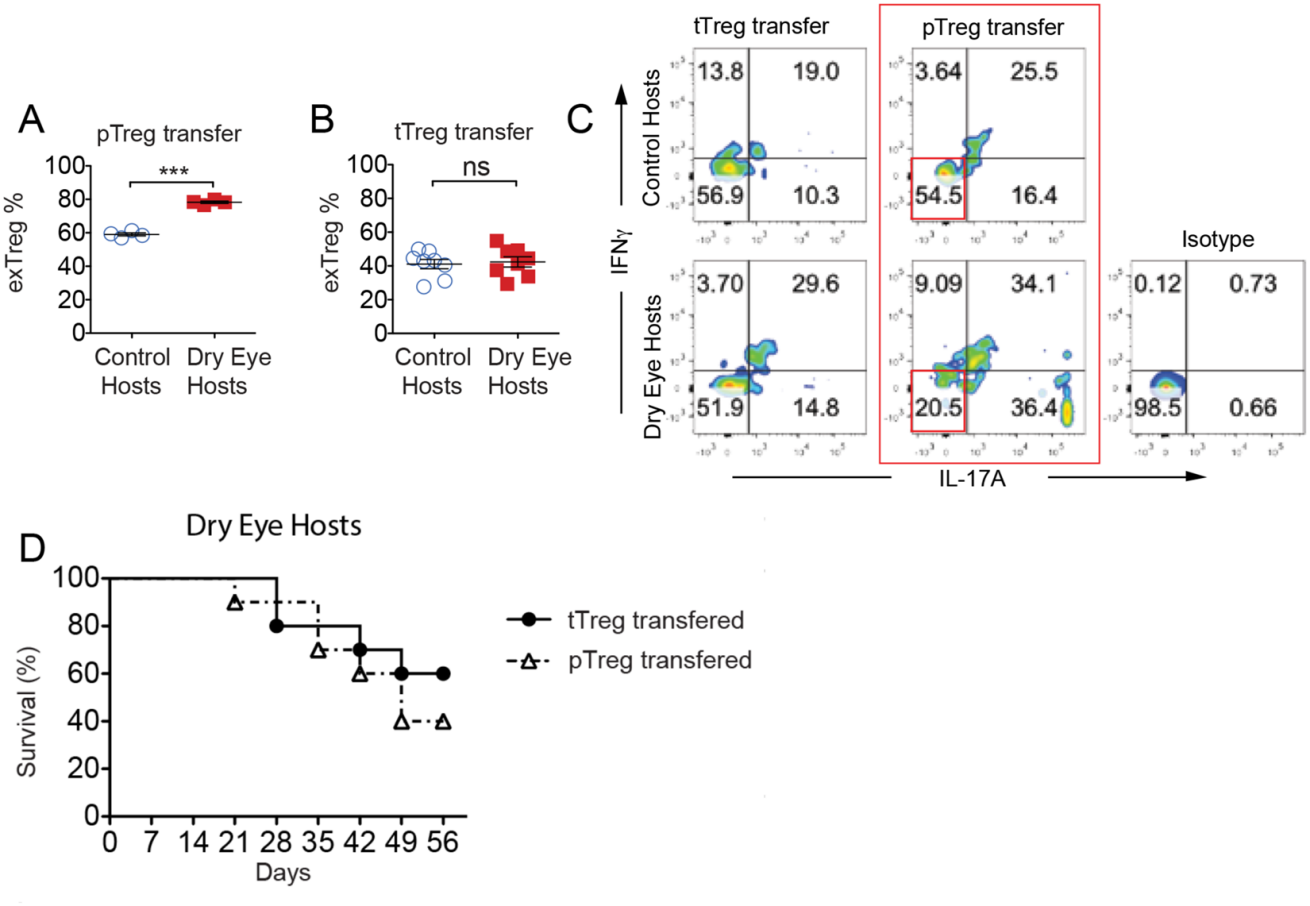
pTregs show a higher susceptibility to convert into exFoxp3 cells after transfer into dry eye hosts. pTregs (CD4^+^CD25^+^Nrp-1^−^) and tTregs (CD4^+^CD25^+^Nrp-1^+^) from transgenic dry eye disease (DED) mice (Foxp3-GFP×R26-RFP) were adoptively transferred into control and DED transplant recipients. (**A** and **B**) Draining lymph nodes were harvested at day 14 post-transplantation. Single cell suspensions were analyzed by flow cytometry. Frequencies of exFoxp3 cells (GFP^−^RFP^+^) from control and DED hosts that received (**A**) pTregs (59.02 ± 0.92% vs. 78.26 ± 0.69%, n = 4, ***p < 0.0001 unpaired two-tailed Student’s t test) or (**B**) tTregs was assessed. (**C**) Representative flow cytometry plots showing frequencies of IL-17 and IFNγ-expressing exFoxp3 cells in draining lymph nodes from control and dry eye hosts. (**D**) Nrp1^+^ tTregs or Nrp1^−^ pTregs from hosts with accepted allografts were adoptively transferred to dry eye hosts, and graft survival was assessed for 8 weeks (n = 10, χ^2^ = 0.6087, p = 0.4353).

To investigate the plasticity of pTregs and tTregs in the context of corneal transplantation, we adoptively transferred Foxp3-GFP×R26-RFP transgenic Nrp-1^−^ pTregs and Nrp-1^+^ tTregs into wild-type control and DED hosts at the time of surgery. Two weeks later, the phenotype of these transferred cells was assessed in dLN using flow cytometry. The frequencies of exFoxp3 (GFP^−^RFP^+^) cells were significantly increased when pTregs were adoptively transferred to DED hosts compared to control hosts (Fig. 3A), suggesting that the inflammatory microenvironment in DED hosts enhanced loss of Foxp3 expression and promoted exFoxp3 formation. However, in the tTreg-transferred mice, no difference was seen in the frequencies of exFoxp3 cells (Fig. 3B). When analyzing the inflammatory cytokine profile of transferred pTregs, we found that the population of Tregs that did not express inflammatory cytokines (i.e. IFN-γ^−^IL-17^−^) was significantly reduced in DED hosts relative to control hosts (20.5% and 54.5%, respectively) (Fig. 3C). In contrast, the difference between the populations of IFN-γ^−^IL-17^−^ Tregs in DED hosts and control hosts following adoptive transfer of tTregs was negligible (56.9% and 51.9%, respectively). Superior corneal allograft survival in hosts receiving adoptive transfer of isolated allosensitized pTregs compared to tTregs has previously been established^13^. To investigate whether the same phenomenon would be observed in DED hosts, we adoptively transferred isolated allosensitized tTregs and pTregs from hosts with accepted allografts to DED hosts receiving fresh allografts. Transfer of pTregs into DED hosts did not confer additional protection from allorejection compared to tTregs (Fig. 3D). These data suggest that the DED microenvironment compromises both the phenotypical and functional stability of transferred pTregs.

### Increased graft rejection observed in DED hosts is mediated via IL-6 and IL-23

**Figure 4:**
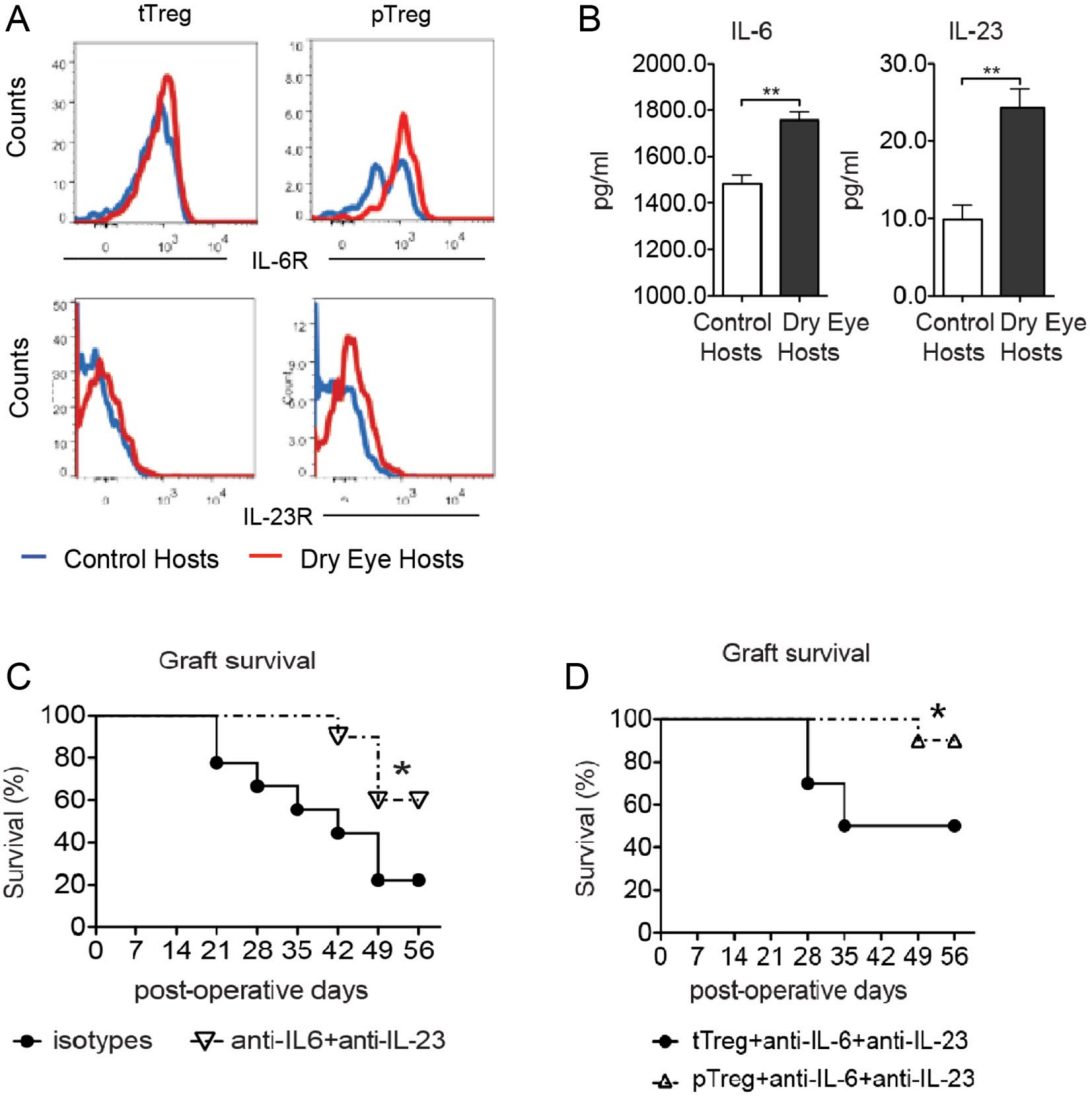
Blockade of IL-6 and IL-23 promotes allograft survival. Draining lymph nodes were harvested 2 weeks post-transplantation. Single cell suspensions were analyzed by flow cytometry. (**A**) Flow cytometry analysis of IL-6R and IL-23R expression by Nrp1^+^ tTregs and Nrp1^−^pTregs from draining lymph nodes of control and DED hosts. (**B**) ELISA analysis showing IL-6 and IL-23 protein levels in the supernatant of explanted draining lymph nodes of healthy control and dry eye (DED) hosts (n = 4, p = 0.0015 and 0.0086, respectively; unpaired two-tailed Student’s t-test). (**C**) Allograft survival of DED hosts treated with anti-IL-6 and anti-IL-23 antibodies or isotype-treated control hosts are shown (n = 10, χ^2^ = 4.525, *p = 0.0334, Log-Rank). (**D**) Allograft survival in DED hosts treated with anti-IL-6 and anti-IL-23 and adoptively transferred with Nrp-1^+^ or Nrp-1^−^ Tregs (n = 10, χ^2^ = 4.503, *p = 0.0338, Log-Rank). Data is presented as mean ± SEM (error bar).

IL-6 and IL-23 signaling are known to play an important role in Treg function^20–22^. In order to evaluate the potential immunomodulation of pTregs and tTregs by IL-6 and IL-23, we examined IL-6 and IL-23 receptor expression by these Treg subsets using flow cytometry. We found that pTregs in DED hosts expressed higher levels of both IL-6R and IL-23R compared to pTregs in control hosts. In contrast, tTregs expressed comparable levels of these receptors in both DED and control hosts (Fig. 4A). Elevated expression of IL-6 and IL-23 has been reported in DED^23,24^. In this study, we also found increased expression of both IL-6 and IL-23 in dLNs of DED hosts (Fig. 4B). To determine the effects of IL-6 and IL-23 *in vivo*, we neutralized these cytokines in DED hosts by intraperitoneal injection of IL-6 and IL-23 neutralizing antibodies, and evaluated allograft survival. Interestingly, while neutralization of IL-6 and IL-23 promoted transplant survival, this approach failed to rescue all transplants in DED hosts (Fig. 4C). To investigate whether both pTregs and stabilizing the microenvironment might establish allotolerance, we adoptively transferred allosensitized pTregs to DED hosts in addition to IL-6 and IL-23 neutralization. This strategy significantly improved graft survival up to 90% (Fig. 4D), suggesting that IL-6 and IL-23 may impede the induction and stability of pTregs, thereby disrupting allotolerance.

## Discussion

By subverting the normal immune privilege of the cornea with desiccating stress prior to transplantation, an inflammatory milieu is created in which allograft survival is jeopardized. Our data demonstrate that Treg frequencies and function are reduced in this setting, and that this corresponds to decreased graft survival. We show that a fraction of Tregs loses Foxp3 expression entirely, becoming IL-17^+^ or IFNγ^+^ exFoxp3 cells that are phenotypically indistinguishable from effector Th17 or Th1 cells, respectively. We investigate the stability of Foxp3 expression in different Treg subsets, demonstrating that neuropilin-1^−^ pTregs are particularly liable to conversion to exFoxp3 cells in response to cues from their microenvironment. Finally, we show that exposure to desiccating stress increases the expression of IL-6 and IL-23 in the dLNs of corneal allograft recipients, and that neutralization of these cytokines promotes transplant survival.

The breadth of CD4^+^ T cell subsets has widened over the past two decades, from the long-established Th1 and Th2 subsets, to a much broader grouping including Th9, Th17, T follicular helper cells, tTregs and pTregs. The evolving paradigm of T cell phenotypic plasticity is based on the hypothesis that these subsets are not fixed, and that T cell subsets can repolarize towards alternative fates in response to environmental changes^1^. Consistent with this paradigm, there is an increasing body of evidence that the Foxp3^+^ Treg lineage is unstable *in vivo*^25^. Moreover, a number of murine studies involving the adoptive transfer of exFoxp3 cells have demonstrated their pathogenicity – specifically their ability to induce experimental autoimmune encephalitis^26^, type 1 diabetes^6^, arthritis^27^, colitis^28^ as well as lung and liver inflammation^29^. Our data further substantiate the pathogenicity of exFoxp3 cells and illustrate their relevance to transplantation immunology.

The prospect of Treg-mediated immunotherapy is the focus of considerable attention^30–33^. In transplantation, strategies to clonally expand Tregs are being explored, in which the suppressive activity of Tregs might be exploited to induce graft tolerance^34^. In the setting of murine corneal transplantation, treatment with low-dose IL-2 has been shown to expand the population of Tregs *in vivo* and increase their immunosuppressive function, resulting in prolonged allograft survival^18^. With Treg-based immunotherapy on the horizon, there is a pressing requirement for clear definitions of Treg subsets, as well as an understanding of the factors that control Treg plasticity. pTregs have been shown to constitute a significant portion of Tregs in the periphery, particularly at mucosal tissues^35^. There is an increasing body of evidence demonstrating that pTregs have a vital and unique role, distinct from tTregs, in maintaining immune homeostasis at these sites^36–38^. There are some important differences in the mechanisms of action of tTregs and pTregs, with polyclonal tTregs modulating Th1 trafficking, and antigen-specific pTregs preventing T-cell priming by controlling the expression of MARCH1 and CD83 on dendritic cells^39^.

Previously, we have shown that tTregs and pTregs have different capacities to promote corneal graft acceptance in high-risk (inflamed and vascularized) host beds^13^. Specifically, we have demonstrated that pTregs from high-risk hosts are reduced in both frequency and function, which correlated with reduced expression of CTLA-4, IL-10 and transforming growth factor-β^13^. Our new data corroborate these findings and provide novel insights into the fate of pTregs in transplantation. Here, we show that transfer of pTregs in an inflamed DED host bed results in a significant increase in frequencies of exFoxp3 cells relative to a healthy uninflamed control host bed - a finding that was not observed in experiments involving the adoptive transfer of tTregs. The level of DNA methylation at Treg-specific demethylated region (TSDR) of the Foxp3 locus has been shown to reflect reduced stability of Foxp3 expression^40^. Furthermore, neuropilin-1^−^ pTregs have been shown to have a higher degree of methylation at the TSDR relative to neuropilin-1^+^ tTregs, corresponding to decreased stability of Foxp3 expression^41^. Our findings are consistent with these observations, suggesting that an inflammatory microenvironment subverts the immunoregulatory capacity of pTregs, with a concomitant loss of Foxp3 expression and conversion to exFoxp3 cells.

Tregs with decreased expression of Foxp3 produce high levels of IL-17^42^, a key cytokine in the pathogenesis of autoimmunity^43^. The interconnected differentiation pathways of Treg and Th17 cells have previously been demonstrated; either subset can develop from FoxP3-RORγt double-positive CD4^+^ T cells depending on signaling from pro-inflammatory cytokines^44,45^. In particular, IL-6 and IL-23 have been shown to have critical roles in altering the balance between Treg and Th17 responses, and can in fact restrain Treg function^20–22,46^. Elevated levels of IL-6 and IL-23 have previously been demonstrated in both murine models of DED and in case-control studies^23,47^. Furthermore, Treg dysfunction has been reported in the recipients of corneal allografts from mice previously exposed to desiccating stress, suggesting that the Th17-driven inflammatory microenvironment of DED may compromise Treg stability^48^. The accumulating data implicating Th17-associated cytokines in Treg plasticity prompted us to examine IL-6 and IL-23 cytokine receptor expression of pTregs and tTregs. Our data show that pTregs in DED hosts express higher levels of both IL-6R and IL-23R relative to tTregs in control hosts. In contrast, tTregs express comparable levels of these receptors in both DED and control hosts. These data illustrate that pTregs have a higher sensitivity than tTregs to IL-6 and IL-23 in an inflamed setting, which may account for their greater propensity for pathological conversion to the exFoxp3 phenotype. By demonstrating a 90% graft survival rate with adoptive transfer of allosensitized pTregs in addition to IL-6 and IL-23 neutralization, we show clearly that modulating the cytokine microenvironment can be an effective strategy to counter the pathological Treg conversion.

In view of the plasticity of Foxp3 expression, it has previously been suggested that a fraction of pathogenic exFoxp3 cells may subsequently revert back to stable Tregs^2^. It is important to note that our lineage-tracing mouse model does not permit differentiation between exFoxp3 cells that have regained Foxp3 expression and Foxp3^+^ Tregs, since both subsets would be GFP^+^RFP^+^. Previous studies using the Foxp3-GFP-Cre-R26-RFP mouse model suggest that cells that transiently express Foxp3 represent a small percentage of the exFoxp3 population, with the majority of exFoxp3 cells being derived from Treg cells with ‘signature’ features (Foxp3^hi^, CD25^hi^, demethylated TSDR)^26,49^.

In summary, this study provides novel insights into Treg phenotypic plasticity in transplantation. By demonstrating increased conversion to exFoxp3 cells in an inflamed host bed, we establish that Treg plasticity in corneal transplantation is moderated by the surrounding microenvironment. Our data show that neuropilin-1^−^ pTregs are particularly prone to conversion to exFoxp3 cells in hosts previously exposed to desiccating stress. We establish that manipulation of Th17-associated cytokines provides a viable method of fostering graft survival in this setting. Further characterization of the cytokine cues that regulate Treg plasticity will provide immunoregulatory strategies for inducing transplantation tolerance.

## Material and Methods

### Animals

Eight- to 10-week old Balb/c and C57/BL6 were purchased from Charles River Laboratories (Wilmington, MA). We crossed transgenic mice expressing a green fluorescent protein–Cre recombinase fusion protein (GFP-Cre) controlled by the Foxp3 promoter on a bacterial artificial chromosome (BAC; Foxp3-GFP-Cre)^50^ and a Rosa26-loxP-Stop-loxP-RFP (R26-RFP) construct-carrying line^17,51^ to generate Foxp3-GFP-Cre-R26-RFP mice. The R26-RFP carrying line was a generous gift from Dr. Hans-Joerg Fehling from the Institute of Immunology, University Ulm, Germany. Mice were housed in a secure, pathogen-free environment in the Schepens Eye Research Institute Animal Facility. All animals were treated in accordance with the Association for Research in Vision and Ophthalmology (ARVO) statement for the Use of Animals in Ophthalmic and Vision Research, and all experiments were approved by the Institutional Animal Care and Use Committee of the Schepens Eye Research Institute.

### Induction of dry eye disease

We induced dry eye disease (DED) by housing mice in a controlled environment chamber for 14 days, as described previously^52^. Briefly, the chamber allows a continuous regulation and maintenance of the temperature (21–23 °C), relative humidity (10–15%), and airflow (15 L/min). Age- and gender-matched control mice were housed in normal environment of the animal facility. During the course of DED induction, we utilized corneal fluorescein staining and the National Institute of Health grading system (NEI, Bethesda, MD) to evaluate corneal epithelial damage caused by desiccating stress^53^. Fourteen days after DED induction, we performed the transplantation procedure, as described below.

### Corneal transplantation and assessment of graft survival

Murine orthotopic corneal transplantation was performed, as previously described^54^. Briefly, a 2-mm central cornea button was excised from a donor mouse; similarly, a 1.5 mm central cornea button was excised from a DED or control (room air) recipient mouse (wild-type BALB/c or transgenic). The donor graft was then secured to the recipient bed with eight interrupted 11–0 nylon sutures (Surgical Specialties Co., Reading, PA). Animals were monitored every 10–15 minutes intraoperatively, and 6, 24, and 72 hours postoperatively for signs of distress. The surgical procedures were performed under anesthesia using intraperitoneal ketamine (120 mg/kg bodyweight) and xylazine (20 mg/kg bodyweight). In addition, subcutaneous buprenorphine (0.1 mg/kg bodyweight) was given intraoperatively and every 8–12 hours up to 48 hours postoperatively to reduce pain. The sutures were removed 7 days post-transplantation. After corneal transplantation, graft survival and opacity were evaluated in a masked fashion weekly for up to 8 weeks using slit lamp biomicroscopy according to a standard opacity-grading system^55^. Grafts with an opacity score of 2 or greater for two consecutive follow-ups were considered as rejected.

### Slit lamp eye examination

Induction of dry eye was confirmed by measuring disruption in the corneal epithelial integrity using corneal fluorescein staining. We applied 0.7 μl of 2.5% fluorescein to the lateral conjunctival sac of the mice; two minutes later, eyes were examined for fluorescein staining with a slit lamp biomicroscope under cobalt blue light. Punctate staining was recorded in a masked fashion using the standard National Eye Institute grading system giving a score from 0 to 3 for each of the five areas of the cornea^53^.

### *In vivo* cytokine neutralization

Lyophilized rat anti-mouse IL-6 and IL-23 neutralizing antibodies and isotype controls (Biolegend, San Diego, CA) were resuspended in PBS to yield a concentration of 1 µg/µl. 100 µg anti-IL-6 and 100 µg anti-IL-23 or isotype controls were administered by intraperitoneal injections every other day for 14 days starting from the day of transplantation. To analyze Treg frequencies, mice were euthanized at day 14, and tissue samples were harvested for further analysis. To determine the effect of IL-6 and IL-23 neutralization on graft survival, mice were treated every other day for 14 days and then every 5 days until day 56, and euthanized thereafter.

### Flow cytometry

Ipsilateral cervical and submandibular draining lymph nodes and corneas were collected from graft recipients 14 days after transplantation and single cell suspensions were prepared, as previously described^56^. In brief, corneas were digested in RPMI media (Lonza, Walkersville, MD) containing 2 mg/ml collagenase type IV (Sigma-Aldrich, St. Louis, MO) and 2 mg/ml DNase I (Roche, Basel, Switzerland) for 60 min at 37 °C, and then filtered through a 70-μm cell strainer. Collected lymph nodes were homogenized in 70-μm cell strainers and single cell suspensions were prepared. Cell suspensions were then washed in fluorescent activated cell sorting (FACS) buffer (2.5% BSA in PBS) and incubated with an anti-Fc-receptor blocking antibody (Clone 91; eBioscience,) to avoid non-specific staining. The following monoclonal antibodies were purchased from BioLegend unless noted otherwise: anti-mouse CD4 (RM4-5), CD25 (PC61), Foxp3 (FJK-16s), IFN-γ (XMG1.2) IL-17A (TC11-18H10.1), Nrp-1 (Clone FAB566N; R&D Systems), IL-6R (D7715A7), and IL-23R (Clone 753317; R&D Systems). For intracellular IL-17A and IFN-γ staining, cells were stimulated in cell culture medium (BD Bioscience) with phorbol 12-myristate 13-acetate (PMA) 50 ng/ml^−1^ and Ionomycin 500 ng/ml^−1^ (Sigma-Aldrich) for 4 hours in the presence of protein transport inhibitor (GolgiStop^TM^). After washing, cells were first stained for surface antigens and then fixed and permeabilized (IC Fixation Buffer and Permeabilization Buffer, eBioscience) before intracellular staining. For intracellular Foxp3 staining, the Foxp3 Staining Buffer Set (Cat. # 00-5523, eBioscience) was used. Isotype-matched control antibodies were used in all experiments. Finally, cells were analyzed by an LSRII flow cytometer (Beckman Coulter), and data were analyzed using FlowJo software X 10.0.7 (Tree Star Inc.).

### *Ex vivo* Treg suppression assay

Responder CD4^+^CD25^−^ T (Tresp) cells were isolated from the dLNs of naïve BALB/c mice using a CD4 T cell isolation kit (Cat. #130-104-454, Miltenyi Biotec), and allogeneic antigen presenting cells (APCs) were isolated from the spleen of naïve C57BL/6 mice by depleting T cells using a CD90.2 isolation kit (Cat. #130-049-101, Miltenyi Biotec). CD4^+^CD25^+^ Tregs were isolated from dLNs of recipients 14 days after transplantation using a commercially available isolation kit (Cat. #130-091-041, Miltenyi Biotec). The phenotype of sorted Tregs was confirmed by assessing their expression of Foxp3 using flow cytometry (purity > 90%). Purified total CD4^+^ Tresp cells (1 × 10^5^) were co-cultured with CD4^+^CD25^+^ Tregs (5 × 10^4^), APCs (1 × 10^5^) and 1 µg/ml anti-CD3 antibody (Clone 145-2C11; R&D Systems) for 72 hours in U-bottom 96-well plate. Bromodeoxyuridine (BrdU) was added to the co-culture at 48 hours. Cell proliferation was measured using the BrdU proliferation kit (Millipore) according to manufacturer’s instructions. Proliferation of CD3-stimulated Tresp cells without adding Tregs was considered as baseline proliferation with 0% suppression. Percent suppression was calculated using the following formula: % suppression = [(Tresp proliferation without Tregs − Tresp proliferation with Tregs)/Tresp proliferation without Tregs] × 100.

### Cell sorting and adoptive transfer

For adoptive transfer experiments, cells were isolated from cervical and submandibular draining lymph nodes and the spleen. These tissues were harvested from room air or DED graft recipients (as indicated), and single cell suspensions were prepared. For isolation of Foxp3^+^ lineage cells and exFoxp3 cells, we used Foxp3-GFP-Cre-R26-RFP double transgenic mice, and collected the draining lymph nodes and spleen to prepare single cell suspensions. Cells were sorted using the BD FACS Aria II sorter at Harvard Stem Cell Institute and The Regenerative Medicine FACS core facility. To sort the subpopulation of tTreg (CD4^+^CD25^+^Nrp-1^+^) and pTreg (CD4^+^CD25^+^Nrp-1^−^), Nrp-1 staining was performed prior to sorting, in combination with CD4 and CD25 staining as indicated in some experiments. The expression of Foxp3 by sorted tTregs and pTregs was confirmed using flow cytometry (purity >90%). For adoptive transfer experiments, 1 × 10^5^ Treg cells/ml were injected to the tail vein of room air or DED recipients.

### Conjunctival and lymph node explant culture with PMA and ionomycin stimulation

For *ex vivo* explant cultures, tissues were harvested 14 days post-transplantation from DED or control hosts. The conjunctiva and all ipsilateral draining lymph nodes were dissected carefully using a pair of sterile vannas scissors and micro-forceps under the surgical microscope. Dissected tissues were then placed in U-bottom 96-well plate in RPMI-100 complete media with additional PMA and Ionomycin for 24 hours; supernatant from each well was collected and analyzed using ELISA.

### Enzyme-linked immunosorbent assay (ELISA)

The supernatant of the tissue explant culture was kept frozen at −80 °C until used for ELISA. ELISA detection kits for murine IL-6 and IL-23 (BMS603HS and BMS6017, eBiosciences) were used according to the manufacturer’s instructions. Absorbance was determined using a POLARstar Optima plate reader (BMG Labtech).

### Statistical analyses

Experiments with more than two groups were analyzed via one-way or two-way analysis of variance (ANOVA) test with post-hoc Tukey’s or Bonferroni’s multiple comparison test. Comparisons between two groups were analyzed using the Student’s t-test. A *P* value of < 0.05 was considered statistically significant. All data are expressed as mean ± SEM. Results are representative of at least three independent experiments. All statistical calculations were performed using Prism Version 5.04 software (GraphPad).

### Data availability

All data generated or analyzed during this study are included in this published article.
